# *Cabp2*-Gene Therapy Restores Inner Hair Cell Calcium Currents and Improves Hearing in a DFNB93 Mouse Model

**DOI:** 10.3389/fnmol.2021.689415

**Published:** 2021-08-19

**Authors:** David Oestreicher, Maria Magdalena Picher, Vladan Rankovic, Tobias Moser, Tina Pangrsic

**Affiliations:** ^1^Experimental Otology Group, InnerEarLab, Department of Otolaryngology, University Medical Center Göttingen, Göttingen, Germany; ^2^Auditory Neuroscience Group, Max Planck Institute of Experimental Medicine, Göttingen, Germany; ^3^Institute for Auditory Neuroscience and InnerEarLab, University Medical Center Göttingen, Göttingen, Germany; ^4^Restorative Cochlear Genomics Group, Auditory Neuroscience and Optogenetics Laboratory, German Primate Center, Göttingen, Germany; ^5^Collaborative Research Center 889, University of Göttingen, Göttingen, Germany; ^6^Multiscale Bioimaging Cluster of Excellence (MBExC), University of Göttingen, Göttingen, Germany

**Keywords:** hair cell, calcium channel, gene therapeutics, adeno-associated virus, hearing, restoration, DFNB93

## Abstract

Clinical management of auditory synaptopathies like other genetic hearing disorders is currently limited to the use of hearing aids or cochlear implants. However, future gene therapy promises restoration of hearing in selected forms of monogenic hearing impairment, in which cochlear morphology is preserved over a time window that enables intervention. This includes non-syndromic autosomal recessive hearing impairment DFNB93, caused by defects in the CABP2 gene. Calcium-binding protein 2 (CaBP2) is a potent modulator of inner hair cell (IHC) voltage-gated calcium channels Ca_V_1.3. Based on disease modeling in *Cabp2^–/–^* mice, DFNB93 hearing impairment has been ascribed to enhanced steady-state inactivation of IHC Ca_V_1.3 channels, effectively limiting their availability to trigger synaptic transmission. This, however, does not seem to interfere with cochlear development and does not cause early degeneration of hair cells or their synapses. Here, we studied the potential of a gene therapeutic approach for the treatment of DFNB93. We used AAV2/1 and AAV-PHP.eB viral vectors to deliver the *Cabp2* coding sequence into IHCs of early postnatal *Cabp2^–/–^* mice and assessed the level of restoration of hair cell function and hearing. Combining *in vitro* and *in vivo* approaches, we observed high transduction efficiency, and restoration of IHC Ca_V_1.3 function resulting in improved hearing of *Cabp2^–/–^* mice. These preclinical results prove the feasibility of DFNB93 gene therapy.

## Introduction

According to the World Health Organization, ∼466 M people worldwide are affected by disabling hearing loss, which can have a big functional, emotional and socio-economic impact on their life, and society. Besides factors like aging or noise, a large proportion of hearing impairment is caused by genetic factors, and has mostly monogenic origins (reviewed in Korver et al., 2017; Wrobel et al., 2021). At present, the treatment of sensorineural hearing loss is limited to the use of conventional hearing aids or, in cases of severe and profound deafness, cochlear implants. Despite their phenomenal success, cochlear implants have their drawbacks. The spread of electric current limits their frequency resolution, which together with a low dynamic range hampers music perception and speech intelligibility in noisy environment. Furthermore, cochlear implants can typically not prevent or stop degeneration of spiral ganglion neurons (SGNs) that is often observed in malfunctioning ears. Thus, novel methods for improved hearing restoration are currently being developed, including gene replacement, and editing or silencing as well as optogenetics (reviewed in Kleinlogel et al., 2020). In the recent years, the potential of a therapeutic approach using AAV-mediated gene transfer has been tested in a few animal deafness models (e.g., Akil et al., 2012, 2019; Jung et al., 2015; Pan et al., 2017; Al-Moyed et al., 2019; Nist-Lund et al., 2019; Rankovic et al., 2021; Taiber et al., 2021).

So far, to our knowledge, no gene-therapy study has targeted the *Cabp2* gene, which encodes calcium binding protein 2, a potent modulator of voltage-gated Ca_V_1.3 channels in inner hair cells (IHCs). Pathologic mutations in *CABP2* have been identified in Iranian, Turkish, Italian, Czech, and Pakistani families (Tabatabaiefar et al., 2011; Schrauwen et al., 2012; Bademci et al., 2016; Marková et al., 2016; Picher et al., 2017; Koohiyan et al., 2019; Park et al., 2020; Safka Brozkova et al., 2020) that lead to autosomal recessive moderate to severe non-syndromic hearing impairment DFNB93. Audiological assessment in those patients revealed an auditory synaptopathy phenotype with often intact otoacoustic emissions signifying outer hair cell (OHC) function being present at least in some patients (Picher et al., 2017). The underlying disease mechanism was recently studied in a knock-out mouse model that suggested enhanced steady-state inactivation of Ca_V_1.3 channels at the IHC ribbon synapse (Picher et al., 2017). Consequently, fewer Ca_V_1.3 channels are available to trigger exocytosis and support IHC neurotransmission, which results in lower temporal precision of sound encoding, and elevated hearing thresholds (Picher et al., 2017; Yang et al., 2018). In contrast to several other deafness mouse models, hair cells of these mice appear to have normal mechanotransduction and synaptic development and show no signs of early secondary degeneration (Picher et al., 2017; the current study; but see Yang et al., 2018), thus suggesting an extended window for gene therapy interventions.

In this study, we tested the potency of two AAV vectors [AAV2/1 and AAV-PHP.eB, abbreviated to PHP.eB in the following, (Chan et al., 2017)] to rescue hearing in *Cabp2^–/–^* mice. AAV containing the coding sequence of wild-type *Cabp2* was injected through a round window into the cochlea of early postnatal mice. Gene transfer by either AAV capsid improved hearing in a majority of 5–8-week-old *Cabp2^–/–^* animals. Furthermore, AAV-mediated expression of Cabp2 restored the typical “non-inactivating” IHC Ca_V_1.3 current properties. This likely underlies partial rescue of hearing and further supports the hypothesis of increased calcium channel inactivation as the key disease mechanism of DFNB93. Collectively, our data suggests that AAV-mediated gene therapy might be a well-suited option to treat DFNB93-related hearing impairment. Normal hair cell development and retained overall hair cell morphology in DFNB93 underline the potential of this approach, and make it particularly interesting for translational approaches in humans.

## Results

**FIGURE 1:**
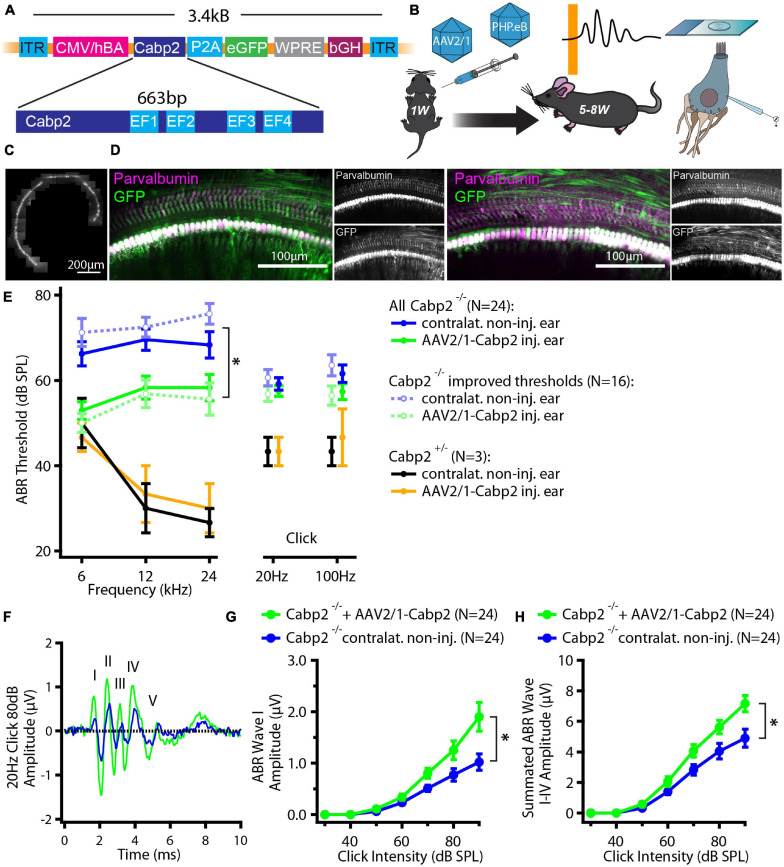
Experimental design and hearing improvement in calcium-binding protein 2 (*Cabp2*)*^–/–^* animals injected with AAV2/1-*Cabp2*. **(A)** Schematic representation of the plasmid construct: An hCMV enhanced hBA promotor with the WPRE enhancer drives the expression of *Cabp2* and *eGFP*, which are separated by a self-cleaving P2A peptide. The construct was inserted in two viral vectors, AAV2/1 and synthetic AAV-PHP.eB. **(B)** Mice of either sex received virus injections at postnatal day P5-7 and were tested for hearing at 5–8 weeks of age. Afterward, cochleae were harvested for patch-clamp experiments or processed for immunohistochemistry. **(C)** Representative image of an apical coil of the organ of Corti from an animal injected with AAV2/1-*Cabp2*-*eGFP* as observed under a fluorescent microscope. Several images were taken and stitched together to reconstruct the whole apical turn. A row of fluorescent IHCs can be observed, indicating high transduction efficiency of the virus. Please note that the fluorescence line of the IHCs is broken three times due to the nylon grid holding the explant in place. **(D)** Representative images of the midcochlear region of two AAV2/1-*Cabp2*-*eGFP* injected cochleae immunolabeled for eGFP and a hair cell marker parvalbumin alpha. A row of transduced IHCs is clearly visible due to strong staining with both antibodies. **(E)** Average ABR thresholds for tone bursts and clicks. Contralateral non-injected ears of each animal served as controls. Out of all 24 injected animals (solid lines; asterisk: two-way RM ANOVA with Šidak’s multicomparisons test; *p* = 0.002, 0.01 and 0.03 for the 6, 12 and 24-kHz tone burst, respectively), 16 showed improved thresholds (dotted lines; two-way RM ANOVA with Šidak’s multicomparisons test; *p* < 0.0001 for all tone bursts). **(F)** Example ABR response to an 80-dB click stimulus (presented at 20 Hz repetition rate) from an injected (green) and the contralateral ear (blue) of the same animal. The ABR waves are marked with roman numbers. **(G)** ABR wave I amplitude in response to 20-Hz clicks of different intensities. **(H)** Summated amplitude of the first four ABR waves in response to 20-Hz clicks of different intensities. Note increased ABR amplitudes in injected *Cabp2^–/–^* ears as compared to contralateral non-injected controls (*p* < 0.001 and < 0.0001 in panel G and H, respectively; asterisks; two-way RM ANOVAs with Šidak’s multicomparisons tests). Asterisks denote *p*-values of less than 0.05.

Hearing restoration using gene therapy offers great hope for causative therapy of genetic forms of severe hearing impairment. Here, we asked whether AAV-mediated gene therapy could be applicable in a knock-out mouse model of non-syndromic hearing impairment DFNB93 (Picher et al., 2017), characterized by moderate to severe hearing loss in patients (Tabatabaiefar et al., 2011; Schrauwen et al., 2012; Bademci et al., 2016; Marková et al., 2016; Picher et al., 2017; Koohiyan et al., 2019; Park et al., 2020; Safka Brozkova et al., 2020). Plasmids encoding for bicistronic expression of Cabp2 and eGFP *via* the P2A peptide driven by the hCMV/hBA promoter and followed by the WPRE and bGH posttranscriptional elements were packaged into AAV2/1, and PHP.eB capsids (Figure 1A and Supplementary Figure 1). Mice received unilateral round window injections at the age of 5–7 days after birth and were tested for hearing, hair cell physiology or cochlear morphology 4–7 weeks afterward (Figure 1B).

We first tested the potency of the AAV2/1 vector carrying the *Cabp2* coding sequence (titer: 2.0 × 10^13^ genome copies/ml) for restoration of hearing in *Cabp2^–/–^* mice. Examination of native eGFP fluorescence in acute cochlear preparations or eGFP immunofluorescence in fixed organs of Corti revealed high transduction efficiency of the AAV2/1 vector (Figures 1C,D and Supplementary Figure 2A) with the large majority of IHCs being eGFP positive. Qualitative inspection of the injected cochleae showed no malformations nor hair cell degeneration, suggesting no gross adverse effects of the viral gene delivery. To evaluate the extent of hearing restoration, we examined auditory brainstem responses (ABRs) to tone bursts and clicks in 5–8-week-old mice (Figure 1E). Hearing thresholds of the injected and non-injected contralateral ears were compared using two-way repeated measures (RM) ANOVA and Šidák’s multiple comparisons test. Over all, the injected ears showed lower thresholds for tone burst frequencies (*p* = 0.002-0.03; *N* = 24 animals). In 67% of the AAV2/1-*Cabp2*-injected animals (16 out of 24 animals), the improvement reached 20 dB SPL or more at least for one of the tested frequencies (*p* < 0.0001 for each tone burst frequency; *N* = 16). Moreover, injected ears showed increased amplitudes of click-evoked ABRs (Figures 1F–H and Supplementary Figure 2; RM ANOVA and Šidák’s multiple comparisons test; *p* < 0.001 for summated as well as individual ABR wave I-IV amplitudes; wave I amplitudes of 1.3 ± 0.2 vs. 0.8 ± 0.1 μV in injected vs. contralateral non-injected *Cabp2^–/–^* ears at 80 dB SPL). Improvement of hearing at least at lower frequencies was also observed when compared to control, non-injected *Cabp2^–/–^* animals, where any potential spread of virus from contralateral injected ears could be excluded (Supplementary Figure 2B; two-way ANOVA, and Šidák’s multiple comparisons test; *p* < 0.03 and 0.001 for 6 and 12 kHz, respectively). Overexpression of Cabp2 in *Cabp2*^+/–^ mice did not alter hearing thresholds, suggesting no adverse effects of viral transduction (Figure 1E).

**FIGURE 2:**
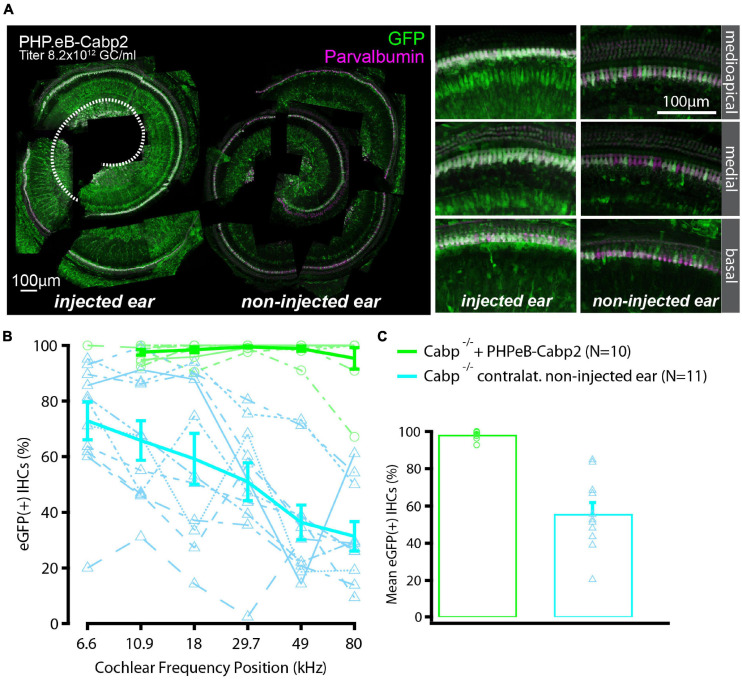
High transduction efficiency of PHP.eB-calcium-binding protein 2 (*Cabp2*) in Cabp2*^–/–^* mice. **(A)** Examples of PHPeB-*Cabp2* injected and contralateral non-injected cochleae immunolabeled for eGFP and parvalbumin alpha [note that the apical portion of the injected side (represented by white dotted line) was used for patch-clamp experiments and is thus missing in the reconstruction]. When using a PHP.eB vector, eGFP expression was also observed in the non-injected contralateral ears albeit at reduced levels. EGFP immunosignal is very strong, but not limited to IHCs (also labeled by parvalbumin alpha). **(B)** Based on eGFP immunofluorescence signal, the transduction efficiency in the IHCs was close to 100% over the entire length of the injected cochleae. In the contralateral ears, the transduction rates were more variable and decreased progressively from the apex toward the base. **(C)** Overall transduction rates were 98 ± 1% (*N* = 10) for injected and 56 ± 6% (*N* = 11) for non-injected cochleae. All values are given as mean ± SEM.

**FIGURE 3:**
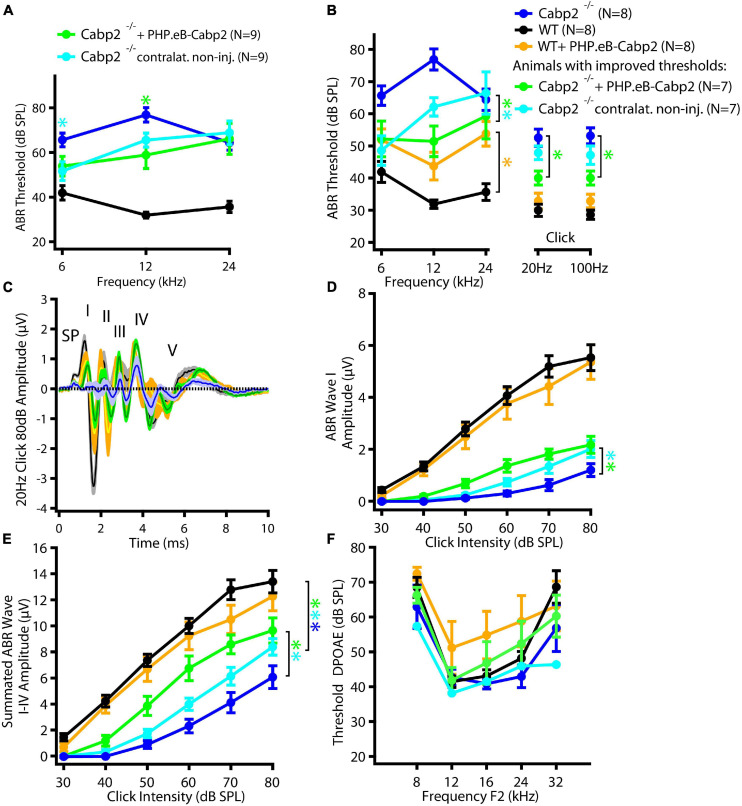
PHPeB-mediated calcium-binding protein 2 (*Cabp2*) gene transfer improves ABR thresholds and amplitude responses in *Cabp2^–/–^* animals. **(A)** Mean ABR thresholds of 5-week-old mice plotted as a function of stimulus frequency for WT (black), *Cabp2^–/–^* controls (blue), and *Cabp2^–/–^* mice injected with PHP.eB-*Cabp2* (injected ears – green; non-injected contralateral ears – cyan). Partial improvement of hearing was observed in injected *Cabp2^–/–^* mice. See Table 1A for statistical analysis. **(B)** Seven out of nine injected *Cabp2^–/–^* animals showed hearing thresholds, which were significantly decreased as compared to non-injected *Cabp2^–/–^* animals (six animals) or on the border of significance (one animal). ABR click responses of these animals are displayed in panels **(C–E)** (see Supplementary Figure 3 for all animals). Overexpression of Cabp2 (or eGFP) in WT animals resulted in increased tone burst ABR thresholds (yellow asterisk). See Tables 1B,C for the statistical analysis. **(C)** Increased average ABR responses to an 80-dB click stimulus (presented at 20 Hz repetition rate) in *Cabp2^–/–^* animals injected with PHP.eB-*Cabp2*. (SP, summating potential; I-IV, ABR waves I-IV) **(D,E)** ABR wave I **(D)** and cumulative ABR wave I-IV amplitudes **(E)** of all groups. PHP.eB-*Cabp2* significantly improved ABR wave amplitudes of *Cabp2^–/–^* mice and did not significantly attenuate amplitude responses in injected wild-type compared to wild-type animals (D - ABR wave I: *p* < 0.0001 for all asterisks except injected ears vs. *Cabp2^–/–^* controls (*p* = 0.001); Tukey’s multiple comparisons test; E – see Table 2 for *p*-values). **(F)** Outer hair cell function was assessed by DPOAEs. Injection of PHP.eB-*Cabp2* did not significantly affect DPOAE thresholds in *Cabp2^–/–^* and a majority of wild-type animals as compared to wild-type controls. On the other hand, DPOAEs were strongly impaired in three out of eight injected wild-type animals. Asterisks denote *p*-, *q*-values of less than 0.05.

To evaluate the impact of the chosen AAV capsids, we then performed the same experiments using the PHP.eB vector (titer: 8.2 × 10^12^ genome copies/ml) that transduces hair cells with high efficiency (Rankovic et al., 2021). Cochleae of injected animals were harvested at 5–6 weeks of age. Preparations were immunostained and eGFP fluorescence levels were analyzed as a proxy of successful transduction (Figure 2). As with the AAV2/1, strong eGFP immunofluorescent signal was detected but not limited to the IHCs (Figure 2A). On average, 98% of IHCs in the injected ears were eGFP positive (Figures 2B,C). Injection of PHP.eB additionally led to transduction of the contralateral non-injected ears albeit with lower efficiency of IHC transduction, declining from the apex toward the cochlear base. ABR recordings revealed lower average hearing thresholds in injected *Cabp2^–/–^* mice when compared to *Cabp2^–/–^* mice that received no viral injections (Figure 3; two-way ANOVA). Partial hearing restoration was found in both, injected and contralateral non-injected ears (Figure 3A, *N* = 9, see Table 1A for statistical analysis). The latter is consistent with the observation of significant hair cell transduction of the contralateral ears when using the PHP.eB vector (Figure 2). Seven out of nine injected animals (78%) displayed ABR thresholds (at least for one tone burst frequency) that were at the border or lower than the mean – 2xSD of the thresholds in the non-injected knock-out control animals (Figure 3B and Table 1B). Furthermore, PHP.eB-mediated expression of Cabp2 increased the amplitudes of click-evoked ABRs (Supplementary Figure 3; Tukey’s multi-comparisons test, *p* < 0.0001; Table 2). This was particularly pronounced in *Cabp2^–/–^* injected mice (Figures 3C–E and Table 2) with improved thresholds (Figure 3B). Partial viral rescue of the wave I amplitude supports the hypothesis of improved auditory function at the level of IHCs/SGNs [2.2 (and 2.0) ± 0.3 vs. 1.2 ± 0.3 μV in injected (and non-injected contralateral) vs. control *Cabp2^–/–^* ears at 80 dB SPL; Figures 3C–E]. To assess cochlear amplification, we recorded distortion product otoacoustic emissions (DPOAEs). These showed comparable thresholds in injected *Cabp2^–/–^* and wild-type control animals, suggesting unaltered OHC function (Figure 3F). Finally, we tested the effects of Cabp2 overexpression in wild-type animals. Using the PHP.eB vector, we observed normal ABR amplitudes (Figures 3C–E, Table 2, and Supplementary Figure 3; 5.4 ± 0.7 and 5.5 ± 0.5 μV in injected, and control *Cabp2^+/+^* animals at 80 dB SPL), but, for unknown reasons, elevated thresholds of tone burst-evoked ABRs compared to non-injected wild-type controls (Figure 3B). The latter finding contrasts our results with the AAV2/1-*Cabp2* in heterozygous animals. Given the lack of antibodies suitable for Cabp2 immunohistochemistry (Picher et al., 2017), we could not directly compare the expression levels of Cabp2 in these animals. However, we suspect that high concentration of Cabp2, which likely occurs in the presence of two functional Cabp2 alleles and with high virus-mediated Cabp2 expression by the PHP.eB vector, may hamper IHC function, e.g., by excessive Ca^2+^-buffering. DPOAE recordings revealed no systematic effect of viral injection in the wild-type animals (Figure 3F). Five out of eight PHP.eB-injected wild-type animals displayed normal DPOAE thresholds (Supplementary Figure 3H). We note that DPOAEs were strongly impaired in three animals despite well preserved ABR click thresholds of 30–40 dB. We can thus not exclude a possibility of a low-mid-frequency OHC defect in a selection of wild-type injected animals. This may be due to mis-expression of (too high concentrations of) Cabp2 in the OHCs, where eGFP was also detected (Figure 2A), or potential complications of the viral injection itself.

**TABLE 1A:** Statistical analysis of ABR thresholds in animals injected with the PHP.eB-calcium-binding protein 2 (*Cabp2*) with the respective controls, including all injected *Cabp2^–/–^* animals.

*p*-value of Student’s *t*-test *p*-value of Dunnett’s test *q*-values of BKY procedure	6 kH tone burst	12 kHz tone burst	24 kHz tone burst	20-Hz click	100-Hz click
injected ears of *Cabp2^–/–^* animals vs. *Cabp2^–/–^* control animals	<0.05* 0.09 0.05*	0.02* 0.005* 0.003*	n.s. 0.9 0.8	n.s. 0.3 0.4	n.s. 0.2 0.3

Contralateral non-injected ears of injected *Cabp2^–/–^* animals vs. *Cabp2^–/–^* control animals	<0.02* 0.035* 0.04*	0.025* 0.1 0.03*	n.s. 0.7 0.8	n.s. 1 0.8	n.s. 0.8 0.6

*Note that the three values in the table cells always represent (from top to bottom): p-value of the Student’s t-test (no correction for multiple comparison), adjusted p-value of Dunnett’s multicomparisons test upon two-way ANOVA, and q-value of the two-stage linear step-up procedure of Benjamini, Krieger, and Yekutieli at the 0.05 level of false discovery rate. The values that may be considered as significant (in case of control of false discovery rates termed discoveries) are marked by asterisks. Depending on the approach (without, or with more or less conservative correction for multiple comparison), the ABR thresholds at 6- and/or 12-kHz tone burst and clicks in injected Cabp2^–/–^ animals (injected and/or contralateral ear) may be viewed as significantly improved/considered true discoveries.*

**TABLE 1B:** Statistical analysis of ABR thresholds in PHP.eB-*Cabp2*-injected animals with the respective controls, including only injected *Cabp2*^–/–^ animals with improved thresholds.

*p*-value of Student’s *t*-test *p*-value of Dunnett’s test *q*-values of BKY procedure	6 kH tone burst	12 kHz tone burst	24 kHz tone burst	20-Hz click	100-Hz click
injected ears of *Cabp2^–/–^* animals vs. *Cabp2^–/–^* control animals	<0.05* 0.03* 0.015*	0.0007* <0.0001* <0.0001*	n.s. 0.5 0.7	0.003* 0.04* 0.02*	0.002* 0.03* 0.018*

contralateral non-injected ears of injected *Cabp2^–/–^* animals vs. *Cabp2^–/–^* control animals	0.01* 0.004* 0.005*	0.005* 0.01* 0.008*	n.s. 0.9 0.7	n.s. 0.6 0.2	n.s. 0.4 0.1

*Note that the three values in the table cells always represent (from top to bottom): p-value of the Student’s t-test (no correction for multiple comparison), adjusted p-value of Dunnett’s multicomparisons test upon two-way ANOVA, and q-value of the two-stage linear step-up procedure of Benjamini, Krieger, and Yekutieli at the 0.05 level of false discovery rate. The values that may be considered as significant (in case of control of false discovery rates termed discoveries) are marked by asterisks. Depending on the approach (without, or with more or less conservative correction for multiple comparison), the ABR thresholds at 6- and/or 12-kHz tone burst and clicks in injected Cabp2^–/–^ animals (injected and/or contralateral ear) may be viewed as significantly improved/considered true discoveries.*

**TABLE 1C:** Statistical analysis of ABR thresholds in wild-type animals injected with the PHP.eB-*Cabp2* with the respective controls.

*p*-value of Student’s *t*-test *p*-value of Šidak’s test *q*-values of BKY procedure	6 kH tone burst	12 kHz tone burst	24 kHz tone burst	20-Hz click	100-Hz click
Wild-type injected vs. non-injected animals	0.05 <0.05* 0.007*	0.01* (w) 0.01* 0.002*	0.0009* <0.0001* <0.0001*	n.s. 1 0.2	n.s. 0.8 0.2

*Note that the three values in the table cells always represent (from top to bottom): p-value of the Student’s t-test (no correction for multiple comparison), adjusted p-value of Dunnett’s multicomparisons test upon two-way ANOVA, and q value of the two-stage linear step-up procedure of Benjamini, Krieger, and Yekutieli at the 0.05 level of false discovery rate. The values that may be considered as significant (in case of control of false discovery rates termed discoveries) are marked by asterisks. Note increased thresholds of tone bursts but not clicks in wild-type injected animals.*

**TABLE 2:** Tukey’s multicomparisons tests for ABR amplitude growth functions, referring to Figure 3E and Supplementary Figure 3.

*p*-values		Combined ABR I-IV waves		Single waves (including all injected animals)			
		All injected *Cabp2^–/–^* animals (*N* = 9)	injected *Cabp2^–/–^* animals with improved thresholds only (*N* = 7)	I	II	III	IV

injected ears of *Cabp2^–/–^* animals vs.	Contralateral non-injected ears of injected	0.009	**	0.6	**	0.2	0.05
	*Cabp2^–/–^* animals						
	
	*Cabp2^–/–^* animals	***	***	0.02	***	***	0.002

Contralateral non-injected ears of injected *Cabp2^–/–^* animals vs.	*Cabp2^–/–^* animals	0.2	0.01	0.5	0.7	0.1	0.8

Wild-type animals vs.	Wild-type injected animals	0.1	>0.06	0.3	***	0.8	0.9

For simplicity, comparisons between different genotypes (irrespective of the treatment) have been omitted from the table. Note that apart from the ABR wave II amplitude in injected wild-type vs. injected Cabp2^–/–^ animals (p = 1), other omitted comparisons show highly significant differences. p < 0.0001 (***), p < 0.001 (**).

**FIGURE 4:**
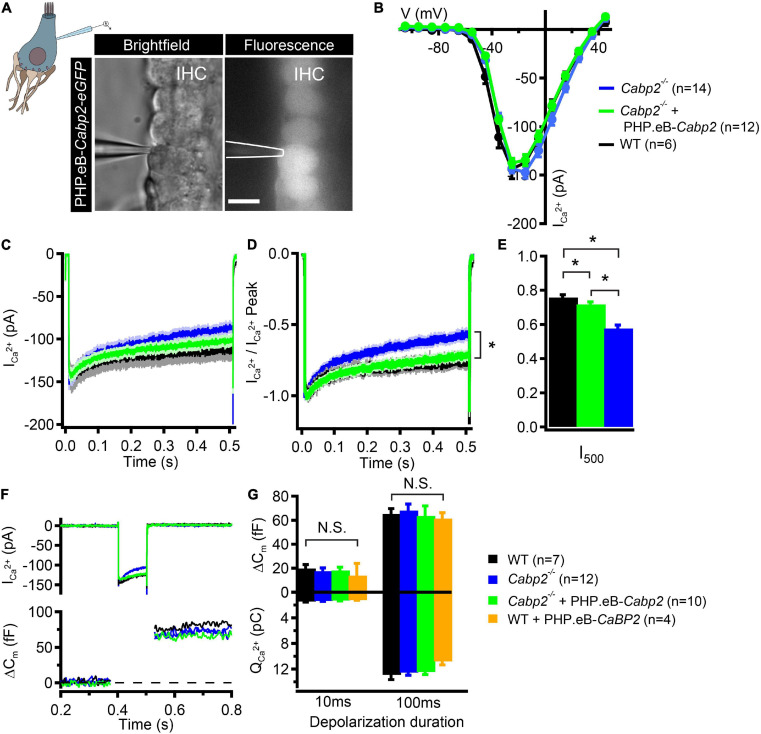
PHP.eB-calcium-binding protein 2 (*Cabp2*)-mediated restoration of IHC Ca_*V*_ 1.3 channel inactivation kinetics. **(A)** Representative brightfield and corresponding fluorescence image showing eGFP-expressing IHCs of a 5-week old *Cabp2^–/–^* animal injected with PHP.eB-*Cabp2*. Scale bar = 10 μm. **(B)** Current-voltage relationships show slightly decreased calcium current amplitudes in IHCs of injected *Cabp2^–/–^* animals. Recordings were performed at 33 ± 0.1°C with 1.3 mM Ca^2+^ in the bath solution. Cells were held at a slightly depolarized voltage of −65 mV. **(C)** Average absolute and **(D)** normalized calcium current responses elicited by 500-ms depolarization steps to the peak calcium current potential. Cabp2-deficient IHCs show significantly enhanced calcium current inactivation that recovers almost completely upon virally induced expression of Cabp2. **(E)** Residual calcium currents after 500 ms of ongoing depolarization (Student’s *t*-test or Wilcoxon rank test; *p* < 0.05; asterisks; see also main text). **(F)** Representative calcium current traces (top) and respective capacitance increments (ΔC_*m*_; bottom) of non-injected *Cabp2*^+/+^, *Cabp2^–/–^* IHCs and IHCs from PHP.eB-*Cabp2*-injected *Cabp2^–/–^* animals upon 100-ms depolarizations to peak calcium current potential. Note that maximal calcium current amplitudes in IHCs from injected *Cabp2^–/–^* animals were 10–15% reduced as compared to non-injected controls. **(G)** Capacitance changes and calcium charge transfer for 10- and 100-ms depolarization steps. No significant differences (N.S.) between the groups were observed. IHCs of PHP.eB-*Cabp2* injected wild-type animals showed a trend toward lower calcium charge transfer and exocytosis. Asterisks denote *p*-values of less than 0.05.

In the last set of experiments, we addressed the function of Cabp2 in modulating IHC Ca_V_1.3 channels by suppressing calcium- and voltage-dependent channel inactivation (Cui et al., 2007; Schrauwen et al., 2012; Picher et al., 2017). We tested whether AAV-mediated delivery of *Cabp2* restores Ca_V_1.3 channel function in the IHCs of *Cabp2^–/–^* animals. For this, we dissected the organs of Corti of the PHP.eB-*Cabp2*-injected *Cabp2^–/–^* animals at 5–6 weeks of age and investigated IHC function using the perforated patch-clamp technique (Figure 4). In order to closely match physiological conditions, recordings were performed at 33.0 ± 0.1°C, at an extracellular calcium concentration of 1.3 mM and holding potential of −65 mV. Using short depolarizations to increasing membrane potentials, we first examined calcium current-voltage relationships in IHCs of different test groups (Figure 4B). In line with a previous study (Picher et al., 2017), calcium currents in Cabp2-deficient IHCs had maximal amplitudes comparable to age-matched controls (Figure 4B), but showed pronounced inactivation upon 500-ms long step depolarizations (Figures 4C–E; *p* = 0.0001 when testing residual currents using Wilcoxon rank test). In IHCs from injected ears of *Cabp2^–/–^* mice, the inactivation of calcium currents was significantly attenuated and residual currents restored to close to wild-type levels (I_500_ values in wild-type IHCs: 76 ± 2%, *Cabp2^–/–^* IHCs: 58 ± 2%, and PHP.eB-*Cabp2*-injected *Cabp2^–/–^* IHCs: 72 ± 1% of initial current; *p* < 10^–5^ and 0.04 for IHCs of PHP.eB-*Cabp2*-injected *Cabp2^–/–^* vs. *Cabp2^–/–^* mice or vs. wild-type mice, respectively; Figure 4E). Finally, we probed exocytosis of *Cabp2^–/–^* IHCs following viral rescue using membrane capacitance recordings. 10- and 100-ms long depolarization steps to maximal calcium current potentials were applied to inspect fast and slow components of exocytosis (example trace in Figure 4F). We found comparable IHC exocytosis in all tested groups (Figure 4G). This implies that the rescue of *Cabp2* expression *via* viral delivery did not interfere with the synaptic physiology of IHCs. However, IHCs of injected wild-type animals showed slightly reduced calcium current amplitudes (Supplementary Figures 4A–C) and consequently a tendency toward decreased calcium charge transfer (Figure 4G). This could be explained by potential harmful effects of Cabp2 overexpression that may reduce the viability of the system. Furthermore, slight adverse effects of viral injections, or of the presence and overexpression of eGFP cannot be excluded. In this respect, it has to be noted that upon inspection of immunohistochemical preparations no hair cell loss was apparent (Supplementary Figure 4D), suggesting that partially elevated ABR thresholds in wild-type injected animals are not due to extensive hair cell death.

## Discussion

In this study, we investigated the potential of an AAV-mediated gene therapeutic approach for DFNB93 hearing impairment. We demonstrate efficient gene delivery with partial rescue of hearing in the *Cabp2^–/–^* mouse model of DFNB93 using two viral capsids, AAV2/1, and synthetic PHP.eB. Both capsids very efficiently transduced IHCs and showed partial improvement of hearing thresholds as well as increased ABR amplitudes in high proportions (ca. 70%) of injected animals. Upon injection of PHP.eB significant transduction along with partial rescue of hearing was also detected in contralateral, non-injected ears. AAV-mediated expression of Cabp2 in the IHCs of *Cabp2^–/–^* animals efficiently inhibited IHC Ca_V_1.3 channel inactivation. Hearing impairment in DFNB93 was suggested to be caused by steady-state inactivation of these channels resulting in hampered synaptic transmission at the first auditory synapse (Schrauwen et al., 2012; Picher et al., 2017). Our data suggests that inhibition of calcium channel inactivation underlies hearing improvement in the injected *Cabp2^–/–^* animals, thereby corroborating the proposed DFNB93 disease mechanism, and provides preclinical proof of concept for future gene therapy of DFNB93.

Both AAV variants partially restored hearing thresholds as well as click-evoked ABR amplitudes in *Cabp2^–/–^* animals. Here, improvement of the ABR wave I amplitudes, which reflect synchronized activity of the SGNs, is of particular importance (see Figures 1F-G, Figures 3C-D and Supplementary Figure 3C). To elucidate the mechanisms underlying heterogeneous effectiveness of these AAV-based rescue approaches, in particular with respect to different frequency ranges, a direct correlation of Cabp2 expression level with IHC physiology, and exact tonotopic position will be required in the future. The results of the current analysis of PHP.eB transduction efficiency and ABR analysis suggest that gene therapy matching the native Cabp2 concentration may be most critical toward the cochlear base. *In situ* hybridization mRNA probes suggested no major concentration differences along the tonotopic axis (Yang et al., 2016), however proper estimation of the Cabp2 protein concentrations in the IHCs in general and along the tonotopic axis more specifically, is still missing. This is relevant, since Cabp2, as other members of the EF-hand protein family, binds calcium ions and may thus act as a calcium buffer (Pangrsic et al., 2015), in particular when present at high concentrations (Picher et al., 2017). Previous work has shown similar endogenous buffer concentration in the apical and basal IHCs of gerbils and rats (Hackney et al., 2005; Johnson et al., 2008), but different coupling of calcium channels to synaptic vesicle fusion (Johnson et al., 2017). Due to looser coupling, exocytosis of the high-frequency basal gerbil IHCs may be more sensitive to calcium buffering as compared to the apical IHCs (Johnson et al., 2017). Hence, basal IHCs may be more susceptible to potentially altered calcium buffering by overexpression of Cabp2. Here, development of promoters and regulatory elements to more closely meet endogenous expression levels of Cabp2 might be of great benefit for the future gene therapeutic studies. Connected to that, our current analysis of the Cabp2 expression was limited to the observation of the fluorescence of eGFP, which is not covalently linked to the protein of interest to not interfere with its functionality. In the future, improved labeling of the Cabp2 (e.g., *via* sensitive and specific Cabp2 antibodies) is required to support further development of the DFNB93 gene therapy.

This is further motivated by the observation that wild-type animals injected with the same viral construct as the *Cabp2^–/–^* animals showed modest elevation of hearing thresholds as well as somewhat reduced IHC calcium currents as compared to non-injected controls. The underlying cause is currently unknown, but may include potential harmful effects of the viral vector, overexpression of a large tracer protein eGFP (Eckrich et al., 2019; but see also Askew et al., 2015; György et al., 2017; Landegger et al., 2017; Suzuki et al., 2017; Dulon et al., 2018; Lee et al., 2020), and the injection procedure itself (Chien et al., 2016) or overexpression of the Cabp2 in either IHCs or other cochlear cell types (e.g., SGNs). The latter would most strongly affect IHCs in injected wild-type animals. For example, in the presence of CaBP2, the Ca_V_1.3 calcium current density in HEK cells may be decreased (e.g., Yang et al., 2006; but see Picher et al., 2017), suggesting a potential role of Cabp2 in regulating Ca_V_1.3 expression levels. Although maximal current amplitudes in IHCs from *Cabp2^–/–^* animals were comparable to wild-type controls, the consequences of Cabp2 overexpression may be stronger. Furthermore, whereas no hair cell loss and no OHC defect was observed in injected *Cabp2^–/–^* animals, DPOAEs were defective in a fraction of injected wild-type animals. Potential reasons include mis-expression of Cabp2 in OHCs and manipulation of the ear during viral injection itself, and could have contributed to elevated tone burst ABR thresholds in these animals. Finally, potential harmful effects of Cabp2 on SGNs or supporting cells have not yet been studied. Further development of more selective promoters for a targeted expression of Cabp2 in IHCs but not OHCs or other cell types may be crucial for the future therapy development.

Re-introduction of Cabp2 in *Cabp2^–/–^* IHCs prevented strong inactivation of Ca_V_1.3 channels typically observed in untreated Cabp2-deficient IHCs. As hypothesized previously, enhanced steady-state Ca_V_1.3 channel inactivation likely hampers IHC synaptic transmission (Picher et al., 2017). We were, however unable to observe reduced exocytic responses in Cabp2-deficient IHCs. As previously (Picher et al., 2017), we hypothesize this could be due to (i) potentially increased extra-synaptic exocytosis due to the loss of Cabp2-dependent calcium-binding capacity as observed in IHCs deficient in mobile calcium buffers (Pangrsic et al., 2015) or to (ii) a failure to mimic *in vivo* conditions (with likely higher IHC activity evoking steady-state inactivation) in the isolated organs of Corti (where calcium channels can recover from inactivation between consecutive stimuli).

The results of the current study, demonstrating that hearing rescue in the Cabp2-deficient animals is possible, are encouraging. However, future studies with improved specificity of viral targeting as well as better control of expression levels of the transgene(s) are required to further improve the extent of hearing rescue. Moreover, a previous study suggested the alternative splice variant *Cabp2*-alt as the most abundant isoform of Cabp2 in the IHCs (Yang et al., 2016). So far, it is not known whether the two long splice variants (*Cabp2*-L, used in this study; and *Cabp2*-alt) differ in their biochemical properties, interactions, or function. A future study should address this question and test whether the choice of the splice variant can affect the extent of hearing rescue. Importantly, *Cabp2^–/–^* animals show normal cochlear development and no early degeneration of stereocilia, ribbon synapses or SGNs, nor hair cell loss (Picher et al., 2017), which suggests a prolonged temporal window for therapeutic intervention in mice and humans may exists, a notion that should be tested in the future. If so, once improved for specificity and with a better dose control, gene therapy holds great promise for success in adult DFNB93 patients.

## Materials and Methods

### Animals

Mice with an exchange of the *Cabp2* exons three and four with the LacZ trapping cassette to obtain global *Cabp2^–/–^* animals (*Cabp2*^LacZ/LacZ^) were used together with the wild-type controls (Picher et al., 2017). All experiments complied with national animal care guidelines and were approved by the University of Göttingen Board for animal welfare and the animal welfare office of the state of Lower Saxony.

### Molecular Cloning of the Construct, Virus Production, and Purification

Viral vectors contained a hybrid hCMV/hBA (human beta actin) promoter to support strong transgene expression of the long isoform of mouse *Cabp2* (*Cabp2*-L; NCBI Reference Sequence: NP_038906.2). A P2A peptide sequence was implemented for bicistronic expression of the Cabp2 and eGFP. For enhanced expression and better stability of the transcript the woodchuck hepatitis virus posttranslational regulatory element (WPRE), and the bovine growth hormone (bGH) polyadenylation sequence were added in the construct. Including the additional regulatory domains the total length of the sequence measured 3.4 kB (Figure 1A). The construct was then packaged into two capsids: AAV2/1 (2.0 × 10^13^ genome copies/ml; purchased from Penn Vector Core, United States) and AAV9-PHP.eB (8.2 × 10^12^ genome copies/ml). PHP-eB particles were generated using our standard AAV purification procedure previously described in a more detail in Huet and Rankovic (2021). In brief, triple transfection of HEK-293T cells was performed using pHelper plasmid (TaKaRa/Clontech), *trans-*plasmid providing viral capsid PHP.eB [generous gift from Viviana Gradinaru (Addgene plasmid #103005^1^ ; RRID:Addgene_103005)], and *cis* plasmid providing wt*Cabp2*-P2A-*eGFP*. PHP.eB viral particles were harvested 72 h after transfection from the medium and 120 h after transfection from cells, and the medium. Precipitation of the viral particles from the medium was done with 40% polyethylene glycol 8,000 (Acros Organics, Germany) in 500 mM NaCl for 2 h at 4°C and then after centrifugation at 4,000 *g* for 30 min combined with cell pellets (already processed by salt-activated nuclease (SAN, Arcticzymes, United States) for additional 30 min SAN incubation at 37°C. Afterward, the cell lysates were clarified by centrifugation at 2,000 *g* for 10 min and then purified over iodixanol (Optiprep, Axis Shield, Norway) step gradients (15, 25, 40, and 60%) at 58,400 rpm for 2.25 h (Zolotukhin et al., 1999; Grieger et al., 2006). Finally, viral particles were concentrated using Amicon filters (EMD, UFC910024) and formulated in sterile phosphate-buffered saline (PBS) supplemented with 0.001% Pluronic F-68 (Gibco, Germany). Virus titers were obtained according to manufacturer’s instructions by determining the number of DNase I resistant vg using qPCR (StepOne, Applied Biosystems) and AAV titration kit (TaKaRa/Clontech). Purity of produced viruses was routinely checked by silver staining (Pierce, Germany) after gel electrophoresis (Novex^TM^ 4–12% Tris-Glycine, Thermo Fisher Scientific) according to manufacturer’s instruction. The presence of viral capsid proteins was positively confirmed in all virus preparations. Viral stocks were kept at −80°C until the day of the experiment. Plasmid vector map is given in Supplementary Figure 1.

### Virus Injections

Mice of either sex were injected at postnatal day P5-7 using the round window approach as described in earlier studies (Huet and Rankovic, 2021; Rankovic et al., 2021). In brief, anesthesia was obtained by isoflurane (5% for anesthesia induction, 2–3% for maintenance, and frequent testing of the absence of hind-limb withdrawal reflex) and analgesia using subdermal injection of buprenorphine (0.1 mg/kg body weight), and carprofen (5 mg/kg body weight, repeated 24 h after procedure) or application of Xylocain (10 mg spray). Body temperature was maintained warm by placing the animal on a remote-controlled custom-build heating blanket. Following a retro-auricular approach, the facial nerve was exposed in order to determine where to puncture the cartilaginous bulla with the injection pipette and target the scala tympani where virus suspension (1–1.5 μl) was injected. Following the injection, the surgical situs was closed by suturing the skin. After injections, 5–8-week-old mice were tested for hearing employing auditory brainstem recordings (ABRs) and distortion product optoacoustic emissions (DPOAEs). Then, the cochleae were processed for electrophysiology and/or immunohistochemistry.

### Systems Physiology

Mice of 5–8 weeks of age were anesthetized by intraperitoneal injections of ketamine (125 mg/kg) and xylazine (2.5 mg/kg), and placed on a heating pad (Hugo Sachs Elektronik – Harvard Apparatus) that kept the body temperature at 37°C. During the measurements, the vitality was checked *via* ECG recordings. A TDT III system (Tucker Davis Technologies) together with BioSig software was used for stimulus generation, presentation and data acquisition. A JBL2402 speaker presented tone bursts at 6, 12, and 24 kHz (10 ms plateau, 1 ms cos^2^ rise/fall) at 40 Hz and clicks of 0.03 ms at 20 Hz or 100 Hz in a free-field configuration. For each measurement, the difference potential between mastoid and vertex was sampled 1,300 times for 20 ms at 50 Hz, amplified 50,000 times and filtered from 400 to 4,000 Hz, to obtain two separate mean ABR traces. The lowest stimulus intensity that resulted in a reproducible waveform in the two traces was considered to represent the ABR threshold value.

Distortion product otoacoustic emissions were measured using the same TDT III system to generate and present stimuli. Two MF-1 speakers presented the primary tones (frequency ratio f2/f1, 1.2; intensity f2 = intensity f1 + 10 dB). Signals were captured with a MKE-2 microphone (Sennheiser), then amplified and digitalized (DMX 6 Fire, Terratec) and analyzed by Fast Fourier Transformation, and a custom written Matlab routine.

### Immunohistochemistry and Confocal Microscopy

Cochleae were fixed in 4% FA on ice for 1 h, rinsed in PBS and decalcified overnight in 120 mM EDTA. After that, Organs of Corti were dissected into 3–4 pieces followed by a blocking step of 1 h in goat serum dilution buffer (GSDB; 16% normal goat serum, 450 mM NaCl, 0.3% Triton X-100, and 20 mM phosphate buffer at pH 7.4) and incubated in primary antibodies in GSDB overnight at 4°C. The following antibodies were used: guinea pig anti-parvalbumin (Cat.-Nr. 195 004; Synaptic Systems), chicken anti-GFP (Cat.-Nr. ab13970; Abcam). Matching Alexa fluorophore conjugated secondary antibodies were then incubated for 1 h at room temperature. Explants were mounted in Mowiol mounting medium and imaged using an Abberior Expert line confocal/STED microscope with a UPlanSAPO 20 × 0.85 NA oil immersion objective, and controlled by Imspector software. For eGFP-fluorescence analysis, organs were processed in parallel and image stacks acquired with the same laser settings, and the pixel size of 800 × 800 nm. Max-projections of image stacks were stitched together by the built-in “*pairwise stitching”* function of ImageJ/Fiji (Schneider et al., 2012). Then, eGFP immunofluorescence was analyzed over the entire length of the cochlea using a custom written Matlab-routine. To estimate the transduction rates, background, unspecific IHC eGFP immunofluorescence was acquired from the cochleae of control, non-injected animal, and processed in the same run as the injected cochleae. Distribution of the eGFP immunofluorescence amplitude in the non-injected control organs was plotted and the value at the 95th percentile taken as the threshold of positive eGFP immunofluorescence. IHCs displaying eGFP immunofluorescence levels above this value were considered as being transduced by the two transgenes.

### Electrophysiology and Fluorescent Imaging

Apical coils from the right (PHP.eB-*Cabp2* injected) cochleae of 5–6 week old mice (P35-42) were dissected in HEPES-HANKS solution which contained (in mM): 10 HEPES, 5.35 KCl, 141.7 NaCl, 0.5 MgSO_4_, 1 MgCl_2_, 11.1 D-glucose, and 3.42 L-glutamine. pH was adjusted to 7.2. The explants were then transferred into a bath solution and kept under constant perfusion at a temperature of 33.0 ± 0.1°C containing (in mM): 111 NaCl, 35 TEA-Cl, 2.8 KCl, 1 CsCl, 1 MgCl_2_, 10 NaOH-Hepes, 11.3 D-Glucose, and 1.3 CaCl_2_ (pH 7.2, 300–310 mOsm). 100 μM apamin (Peptanova, Germany) was added to the solution to block any potential remaining SK2 channels. The pipette solution contained the following (in mM): 137 mM Cs-gluconate, 10 mM TEA-Cl, 10 mM 4-AP, 10 mM Cs-HEPES, 1 mM MgCl2, and 300 μg/ml amphotericin B (pH 7.2 and 290 mOsm). An EPC-9 amplifier controlled by Pulse software (HEKA Elektronik, Germany) was used for membrane current and capacitance recordings from the IHCs. The holding potential was set to −65 mV. The recordings of calcium currents and exocytosis in perforated-patch configuration were performed, and analyzed as described before (Moser and Beutner, 2000). Calcium currents were sampled at 50 kHz, low-pass filtered at 2.9 kHz and leak corrected using a P/N-protocol. Voltage was corrected for liquid junction potential of 15 mV. Fluorescent imaging of the eGFP signal was performed using a narrow band light source (Polychrome IV, Till Photonics, Germany) at a wavelength of 488 nm and a CCD camera (Till Photonics, operated by Till-Vision software). Images were taken with an exposure time of 200 ms.

### Data and Statistical Analysis

Data analysis was performed using MATLAB (Mathworks), Igor Pro (Wavemetrics), and ImageJ (NIH) software. Means and grand averages are expressed as ±SEM. Statistical analysis was performed using the Igor Pro and Prism (GraphPad). The Jarque–Bera and F test were used to determine whether the samples have normal distribution, and equal variance. These tests were then followed by two-tailed Student’s *t*-test, or—when data were not normally distributed and/or variance was unequal between samples—the Mann–Whitney–Wilcoxon test for statistical comparisons between two samples. For matched comparisons of the ABR data in AAV2/1-injected animals two-way RM ANOVA, followed by Šidak’s multicomparisons test was performed. ABR thresholds in PHP.eB-injected animals were inspected using Student’s *t*-tests, and two-way ANOVA with a correction for the multiple comparisons by Dunnett’s multicomparisons tests and by controlling the false rate discovery (FRD) using the two-stage linear step-up procedure of Benjamini, Krieger, and Yekutieli at the 0.05 level of FRD. For ABR wave analysis Tukey’s multicomparisons tests were applied.

## Data Availability Statement

The raw data supporting the conclusions of this article will be made available by the authors, without undue reservation.

## Ethics Statement

The animal study was reviewed and approved by the board for animal welfare of the University Medical Center Göttingen and the animal welfare office of the state of Lower Saxony (LAVES; Animal protocol numbers 14.1391, 19.3133, and 19.3134).

## Author Contributions

TP, DO, MP, and TM designed the study. DO performed experiments (cell electrophysiology, immunohistochemistry, and part of ABR/DPOAE recordings) and analyzed the data. MP and VR designed the plasmids. VR generated PhP.eB viral vectors. MP and TP performed initial experiments. MP performed molecular cloning. TP analyzed the data. TM established inner ear injections. TP and DO wrote the manuscript. All authors revised the manuscript.

## Conflict of Interest

The authors declare that the research was conducted in the absence of any commercial or financial relationships that could be construed as a potential conflict of interest.

## Publisher’s Note

All claims expressed in this article are solely those of the authors and do not necessarily represent those of their affiliated organizations, or those of the publisher, the editors and the reviewers. Any product that may be evaluated in this article, or claim that may be made by its manufacturer, is not guaranteed or endorsed by the publisher.

## Footnotes

1
http://n2t.net/addgene:103005

## Supplementary Material

Supplementary Figure 1 Calcium-binding protein 2 (*Cabp2)*-P2A-*eGFP* construct assembly and pAAV vector used for AAV packaging of *Cabp2*-P2A-*eGFP*. pAAV *cis-*plasmid providing *Cabp2* and *eGFP* with a P2A peptide sequence for bicistronic expression of the two proteins. Asterisks denote impaired ability of the restriction enzyme to cleave at the restriction site.

Supplementary Figure 2 ABR responses and eGFP expression in AAV2/1-*Cabp2*-injected animals. **(A)** Reconstructions of native eGFP fluorescence of AAV2/1-*Cabp2*-injected organs of Corti observed under a fluorescence microscope. An almost continuous line of eGFP fluorescence in IHCs is observed. Please note that the fluorescence can not be observed where a nylon grid crosses the explants. **(B)** Mean ABR thresholds of AAV2/1-*Cabp2*-injected *Cabp2^–/–^* vs. non-injected *Cabp2^–/–^* animals. Hearing was improved for 6 and 12-kHz tone bursts (two-way ANOVA with Šidak’s multicomparisons test). **(C)** ABR wave II-V amplitudes in injected and contralateral, non-injected ears of AAV2/1-*Cabp2*-injected *Cabp2^–/–^* animals to an 80-dB 20-Hz click stimulus. Note increased amplitudes of injected ears for waves II, III, and IV (Šidak’s multicomparisons test, *p* < 0.001 for wave II and III, and < 0.0001 for wave IV). Asterisks denote *p*-values of less than 0.05.

Supplementary Figure 3 20-Hz click ABR amplitudes including all PHP.eB-calcium-binding protein 2 (*Cabp2*)-injected animals. **(A)** Average responses to an 80-dB click stimulus presented at the repetition rate of 20 Hz. **(B)** Combined wave I-IV and **(C–G)** single ABR wave amplitudes to an 80-dB 20 Hz click stimulus of all animals. Note increased amplitudes from injected *Cabp2^–/–^* ears as compared to control *Cabp2^–/–^* animals for waves I, II, III, and IV (Tukey’s multicomparisons test, see Table 2 for *p*-values). ABR wave amplitudes in injected *Cabp2*^+/+^ animals were comparable to the non-injected *Cabp2*^+/+^ controls. **(H)** DPOAE thresholds in the individual injected and non-injected *Cabp2*^+/+^ animals. Asterisks denote *p*-values of less than 0.05.

Supplementary Figure 4 The effects of calcium-binding protein 2 (Cabp2) overexpression on the IHC calcium currents and morphology in wild-type animals. **(A)** Current-voltage relationship in IHCs of injected wild-type animals vs. non-injected controls. **(B,C)** Average absolute **(B)** and normalized **(C)** calcium current responses upon 500-ms depolarization steps to the peak calcium current potential in injected and non-injected wild-type IHCs. Overexpression of Cabp2 (or eGFP) results in partially reduced calcium current amplitudes. **(D)** Representative example of PHPeB-*Cabp2* injected cochlea of a wild-type animal immunolabeled for eGFP and parvalbumin alpha. No significant loss of hair cells was observed upon viral injection and overexpression of Cabp2 and eGFP.
