# Supplementary figures and images for: Cabp2-Gene Therapy Restores Inner Hair Cell Calcium Currents and Improves Hearing in a DFNB93 Mouse Model
Source: Front Mol Neurosci. 2021 Aug 19;14:689415. doi: 10.3389/fnmol.2021.689415 (PMC8417311; doi:10.3389/fnmol.2021.689415)

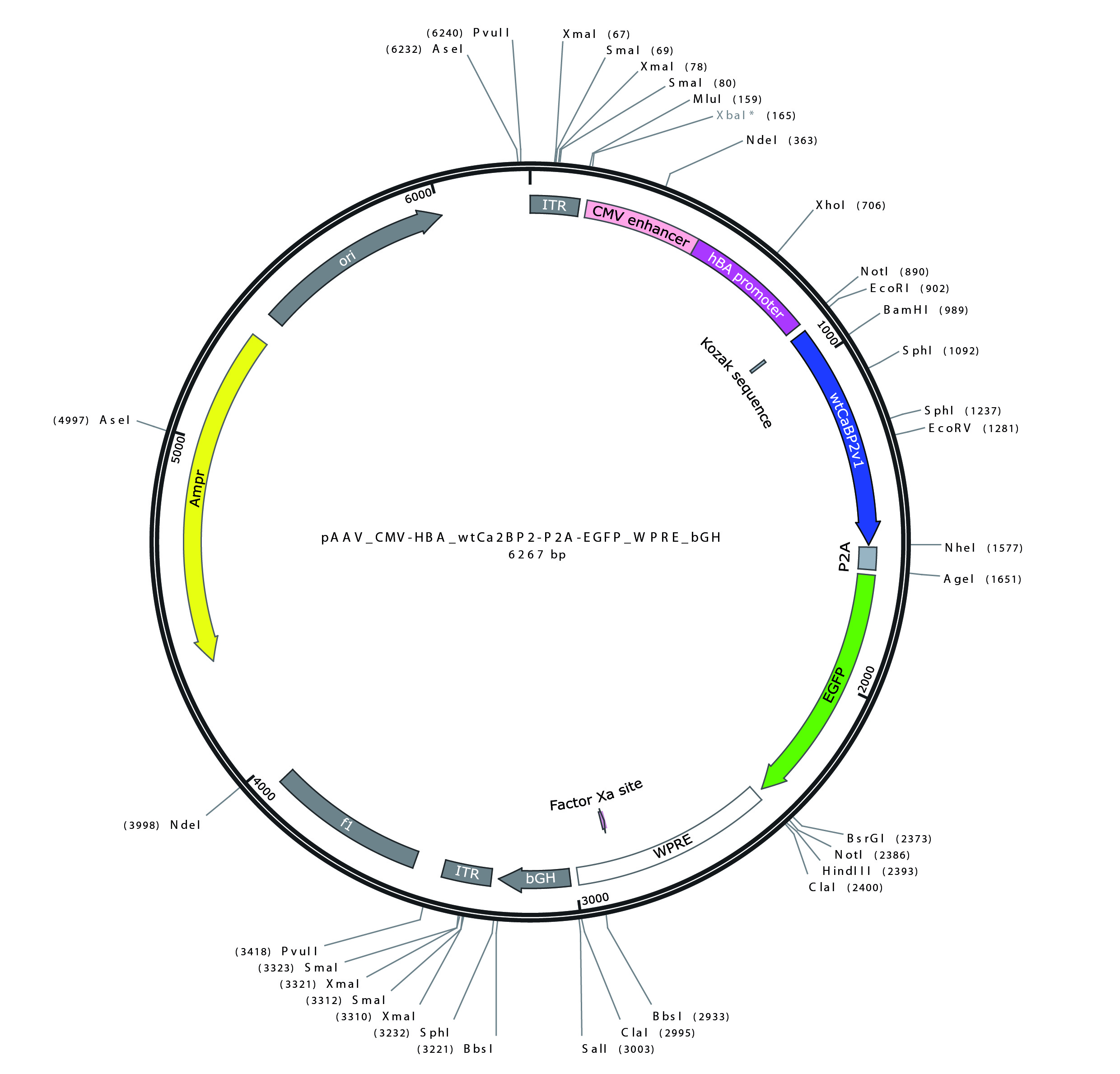

Supplement: Supplementary Figure 1 — Calcium-binding protein 2 (Cabp2)-P2A-eGFP construct assembly and pAAV vector used for AAV packaging of Cabp2-P2A-eGFP. pAAV cis-plasmid providing Cabp2 and eGFP with a P2A peptide sequence for bicistronic expression of the two proteins. Asterisks denote impaired ability of the restriction enzyme to cleave at the restriction site. [file Image_1.jpg]

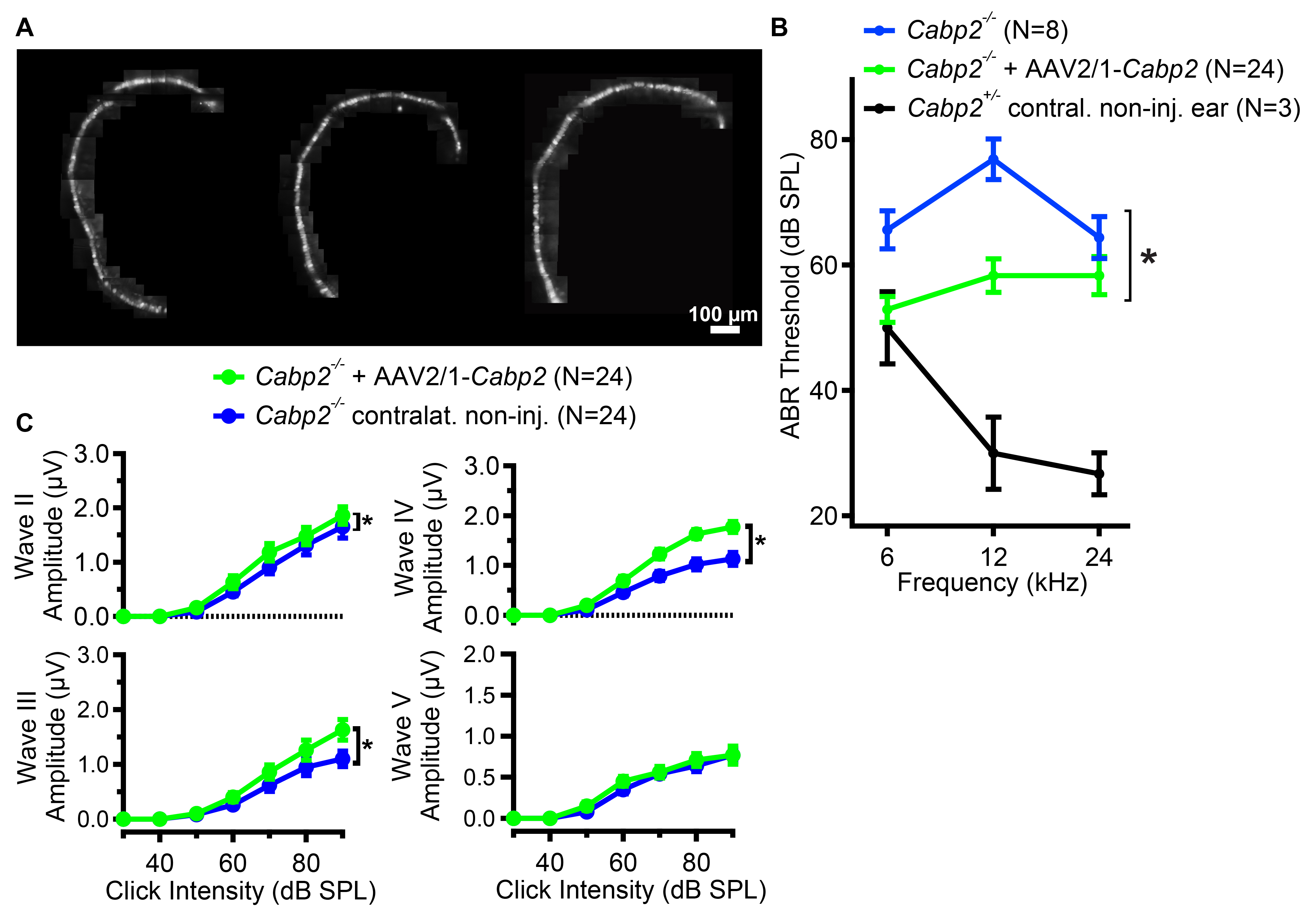

Supplement: Supplementary Figure 2 — ABR responses and eGFP expression in AAV2/1-Cabp2-injected animals. (A) Reconstructions of native eGFP fluorescence of AAV2/1-Cabp2-injected organs of Corti observed under a fluorescence microscope. An almost continuous line of eGFP fluorescence in IHCs is observed. Please note that the fluorescence can not be observed where a nylon grid crosses the explants. (B) Mean ABR thresholds of AAV2/1-Cabp2-injected Cabp2–/– vs. non-injected Cabp2–/– animals. Hearing was improved for 6 and 12-kHz tone bursts (two-way ANOVA with Šidak’s multicomparisons test). (C) ABR wave II-V amplitudes in injected and contralateral, non-injected ears of AAV2/1-Cabp2-injected Cabp2–/– animals to an 80-dB 20-Hz click stimulus. Note increased amplitudes of injected ears for waves II, III, and IV (Šidak’s multicomparisons test, p < 0.001 for wave II and III, and < 0.0001 for wave IV). Asterisks denote p-values of less than 0.05. [file Image_2.jpg]

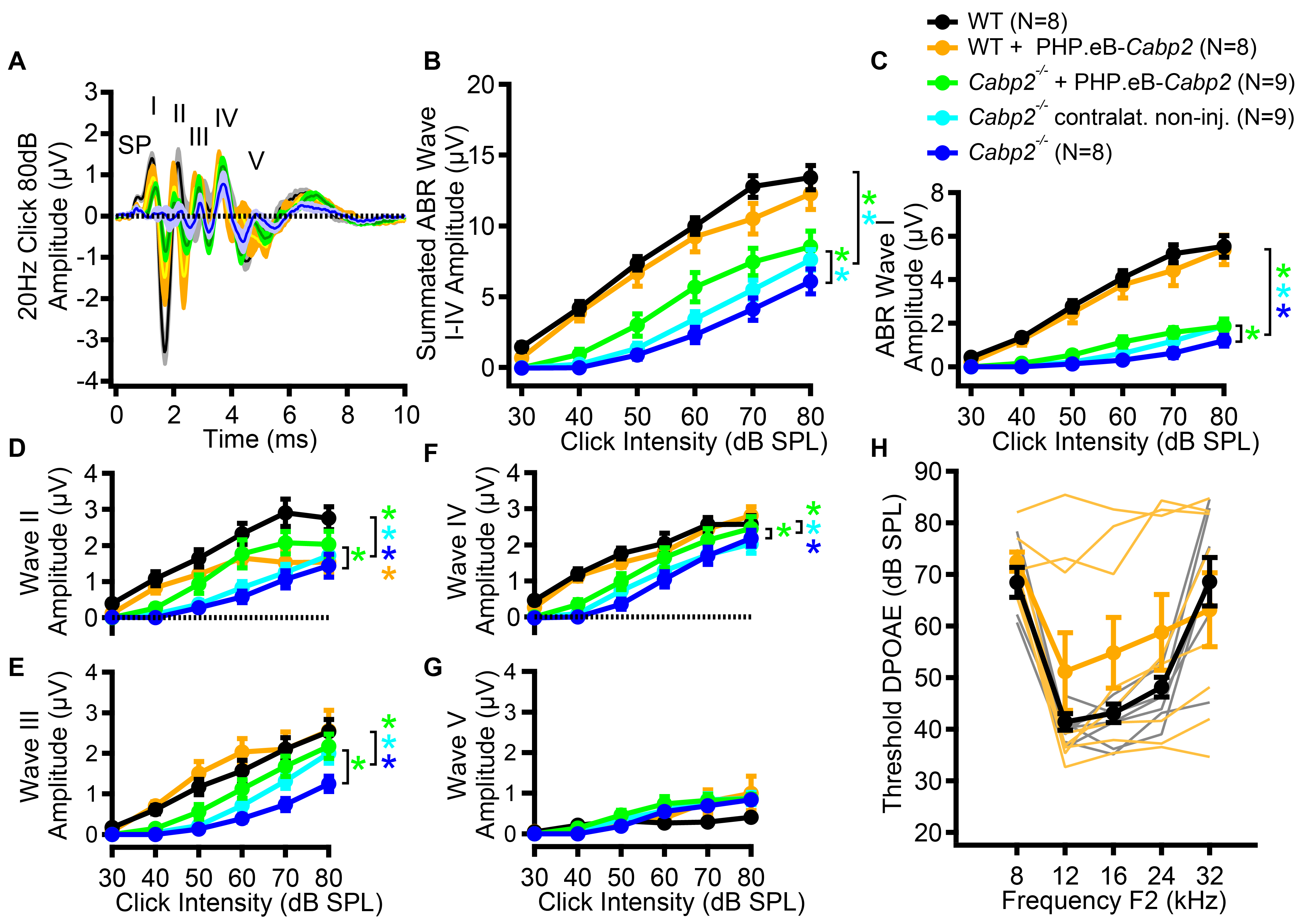

Supplement: Supplementary Figure 3 — 20-Hz click ABR amplitudes including all PHP.eB-calcium-binding protein 2 (Cabp2)-injected animals. (A) Average responses to an 80-dB click stimulus presented at the repetition rate of 20 Hz. (B) Combined wave I-IV and (C–G) single ABR wave amplitudes to an 80-dB 20 Hz click stimulus of all animals. Note increased amplitudes from injected Cabp2–/– ears as compared to control Cabp2–/– animals for waves I, II, III, and IV (Tukey’s multicomparisons test, see Table 2 for p-values). ABR wave amplitudes in injected Cabp2+/+ animals were comparable to the non-injected Cabp2+/+ controls. (H) DPOAE thresholds in the individual injected and non-injected Cabp2+/+ animals. Asterisks denote p-values of less than 0.05. [file Image_3.jpg]

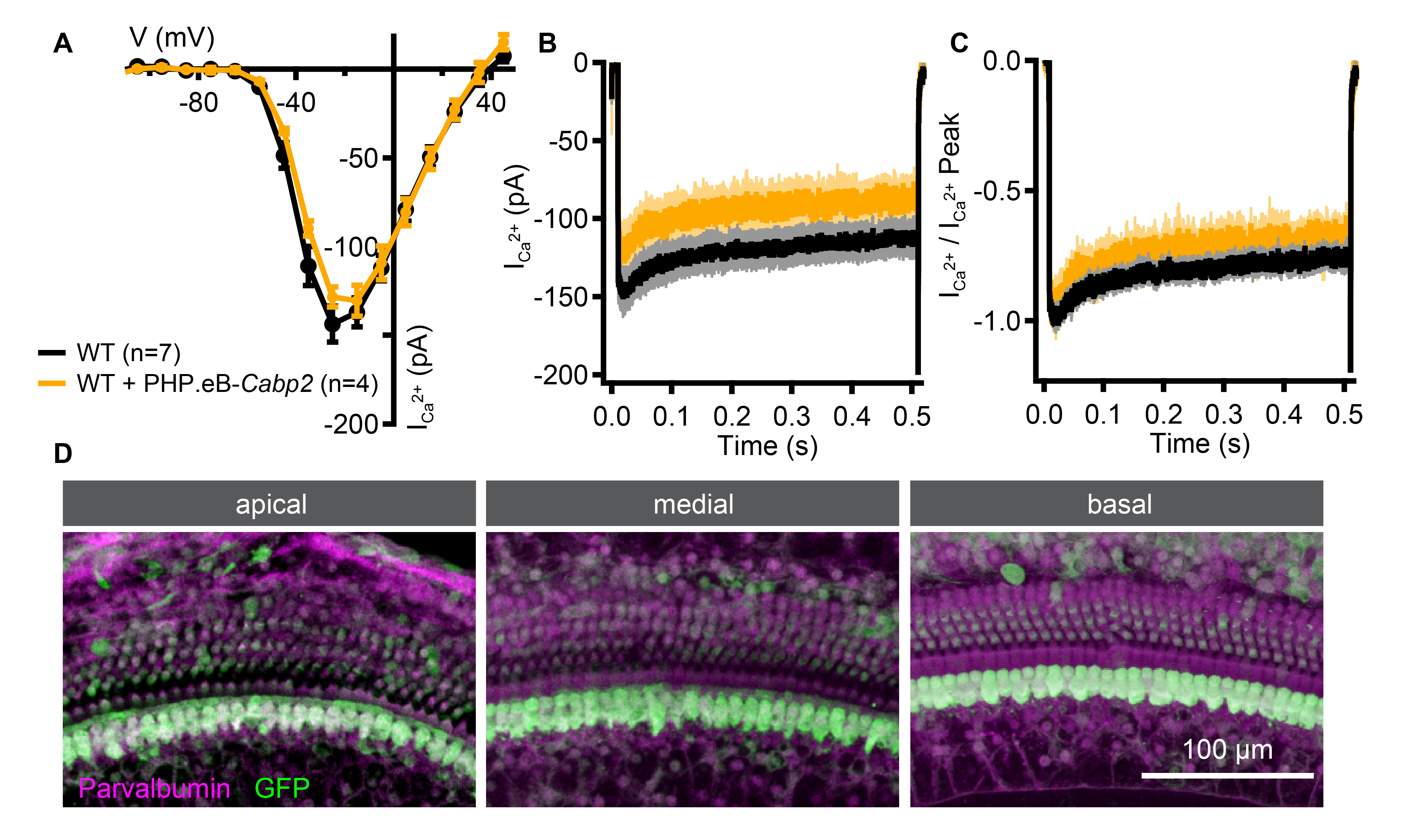

Supplement: Supplementary Figure 4 — The effects of calcium-binding protein 2 (Cabp2) overexpression on the IHC calcium currents and morphology in wild-type animals. (A) Current-voltage relationship in IHCs of injected wild-type animals vs. non-injected controls. (B,C) Average absolute (B) and normalized (C) calcium current responses upon 500-ms depolarization steps to the peak calcium current potential in injected and non-injected wild-type IHCs. Overexpression of Cabp2 (or eGFP) results in partially reduced calcium current amplitudes. (D) Representative example of PHPeB-Cabp2 injected cochlea of a wild-type animal immunolabeled for eGFP and parvalbumin alpha. No significant loss of hair cells was observed upon viral injection and overexpression of Cabp2 and eGFP. [file Image_4.jpg]
